# Dissemination of Acupuncture-Moxibustion Clinical Practice Guidelines among Clinical Practitioners: A Systematic Review of Quality Assessment Studies

**DOI:** 10.1155/2022/8334397

**Published:** 2022-04-07

**Authors:** Ying Wang, Qin Wang, Yalan Peng, Yonggang Zhang, Nian Li

**Affiliations:** ^1^Department of Medical Administration, West China Hospital, Sichuan University, Chengdu 610041, China; ^2^Department of Evidence-Based Medicine and Clinical Epidemiology, West China Clinical School of Medicine, Sichuan University, Chengdu 610041, China; ^3^Hospital Infection Management Department, West China Hospital, Sichuan University, Chengdu 610041, China; ^4^Department of Periodical Press, West China Hospital, Sichuan University, Chengdu 610041, China; ^5^Chinese Evidence-Based Medicine Center, West China Hospital, Sichuan University, Chengdu 610041, China

## Abstract

Acupuncture clinical practice guidelines are authoritative medical recommendations developed by evaluating and integrating acupuncture-related evidence. However, their synthesis and dissemination are not integrated, and clinical practitioners require more credible effective evidence. The study aim was to systematically review problems disseminating acupuncture clinical practice guidelines to clinical practitioners, to facilitate evidence dissemination. This systematic review included searches of PubMed, EBSCO, Web of Science, and four major Chinese electronic databases (CNKI, VIP, Wanfang Database, and SinoMed) from inception to October 26, 2021. Two independent reviewers screened the literature, extracted information, and evaluated the quality of included studies. A systematic review was subsequently performed. Eleven studies were reviewed: nine (81.8%) cross-sectional surveys and two (18.2%) systematic reviews. The evaluated clinical practice guidelines differed across studies; seven studies (63.6%) evaluated guidelines for a specific disease, one (9.1%) evaluated guidelines for acupuncture therapies (e.g., moxibustion and fire acupuncture), one (9.1%) evaluated US acupuncture guidelines and recommendations, and two (18.2%) did not describe the guideline content. The included studies used different evaluation indicators. Guideline dissemination problems included lack of guideline standardization, unclear target population, mismatch between guidelines and application environment, lack of reliable health economics evaluation, poor quality content of the recommendations, lack of linkage between recommendations and evidence, and disassociation of recommendations from clinical practice et al. The development and publishing of credible acupuncture clinical practice guidelines is urgently needed to improve the usability of guidelines and standardize and disseminate tools for analysing information to clinical practitioners and to help the domestic and international acupuncture community to apply evidence to practice. Recommendations for promoting the dissemination of acupuncture clinical practice guidelines are to define clinical events suitable for the target population, to develop recommendations relevant to clinical practice, to improve the evidence evaluation index system, and to further standardize the method and process of formulating guidelines.

## 1. Introduction

Acupuncture-moxibustion is a method of preventing and treating various diseases [1–4]. Numerous clinical studies have provided good evidence that acupuncture-moxibustion has effects on respiratory [5], gastrointestinal [6], neurological diseases [7, 8], and pain [9]. With the development of Chinese medicine, acupuncture-moxibustion is now widely used in more than 180 countries [10]. The application of acupuncture-moxibustion is no longer determined solely by the personal experience of the physician but is supported by scientific evidence. For example, the Guideline for Clinical Practice of Acupuncture-Moxibustion (group standards) [10], developed and published by the Chinese Acupuncture and Moxibustion Society, comprises authoritative medical recommendations developed by systematically evaluating and integrating evidence related to acupuncture-moxibustion, considering the balance of pros and cons, then making the most appropriate recommendations.

Clinical practitioners need effective ways to obtain more credible evidence to ensure that patients receive effective diagnosis, treatment, and follow-up. These measures should be decided by a consensus between practitioners and patients and should be tailored to each patient's specific needs. However, links in the evidence chain, which includes production, synthesis, dissemination, and implementation, are not currently integrated [11]. There remains a large gap between the production/synthesis of evidence and its dissemination and use among clinical practitioners [12] as a basis for clinical decision-making or policy development. This poses a key challenge to the clinical efficacy of acupuncture-moxibustion. Systematic evaluation is needed to consider the quality of evidence, the pros and cons of different interventions, and patients' preferences and costs. This would help to produce clinical practice guidelines that provide optimal recommendations and disseminate evidence to clinical practitioners [13].

Studies on problems with the dissemination of acupuncture-moxibustion clinical practice guidelines to clinical practitioners have increased; however, such studies use inconsistent methods, tools, participants, and outcomes [13]. This systematic review re-evaluated the literature on relevant assessment tools and research methods (e.g., AGREE II, questionnaires, and Delphi method) to assess the problems with clinical guideline dissemination to clinical practitioners. We summarized and analyzed the quality of clinical practice guidelines and reporting standards, clinical practitioners' attitudes and views toward guidelines, problems encountered during the use of the guidelines, and factors that affect the use of acupuncture-moxibustion guidelines in clinical practice. We present suggestions for future development of acupuncture-moxibustion clinical practice guidelines to address current problems and obstacles in guideline dissemination and clinical practice. We hope that this will help to improve the quality of evidence, disseminate evidence, help clinical practitioners to make appropriate decisions about clinical problems, improve the clinical efficacy of acupuncture-moxibustion, reduce medical costs, and promote the rapid development of acupuncture-moxibustion.

## 2. Materials and Methods

### 2.1. Registration

The current systematic review is part results of a systematic review, and the systematic review registration [CRD42021279104] was completed on October 17, 2021.

### 2.2. Search Strategy

This systematic review included searches of Chinese and English articles in PubMed, EBSCO, Web of Science, and four major Chinese electronic databases (CNKI, VIP, Wanfang Data, and SinoMed) from inception to October 26, 2021 (Appendix 1). Additional search of unpublished literature (including conference papers, master's and doctoral theses, and gray literature) was also conducted.

### 2.3. Inclusion and Exclusion Criteria

#### 2.3.1. Inclusion Criteria

Inclusion criteria were as follows: studies using various methods/tools for quality assessment of dissemination and application of clinical practice guidelines for acupuncture-moxibustion in clinical practice.

#### 2.3.2. Exclusion Criteria

Exclusion criteria were as follows: literature in languages other than English and Chinese; duplicate publications; publications for which the data could not be obtained, such as conference abstracts; clinical practice guidelines; literature related to the interpretation of guidelines, consensus, standards, procedures, and norms; studies on acupuncture-moxibustion methodology in addition to acupuncture-moxibustion guidelines; and studies on acupuncture-moxibustion service processes.

### 2.4. Literature Screening

Two researchers independently screened the literature and cross-checked the data. In the event of disagreement, a third researcher was invited to adjudicate. The literature screening was carried out by first reading each source's title and abstract. After excluding obviously irrelevant sources, the full text was read to determine the final inclusion. Literature management and duplication removal were performed using NoteExpress 3.4.0.8878 software.

### 2.5. Data Extraction

The literature was independently extracted and cross-checked by 2 researchers. Disagreements were resolved by discussion, and a consensus was reached through a third researcher. For sources with incomplete information, the authors of the source were contacted if possible. Data were extracted using a predeveloped data extraction form. Before performing the formal extraction, the researchers involved received training and participated in a practice extraction exercise. The extracted data mainly included baseline characteristics: authors, year of publication/update, country or region, study design, study population, study institution, and publication journal. The guideline evaluation examined evaluation subjects, evaluation/methodological tools, evaluators, problems identified, and recommendations made.

### 2.6. Quality Assessment of Included Literature

Because no specific checklist has been developed to assess the quality of articles evaluating acupuncture-moxibustion guidelines, we referred to the Joanna Briggs Institute (JBI) checklist for evaluating cross-sectional studies [14] to evaluate the quality of included cross-sectional studies. The following evaluation criteria were used: 1. Were the criteria for inclusion of evaluators clearly defined? 2. Were the guidelines and settings studied described in detail? 3. Was the evaluation of the guidelines performed in a valid and reliable manner? 4. Was the situation measured using objective criteria? 5. Were confounding factors affecting the practice of the guidelines identified? 6. Were strategies in place to address confounding factors? 7. Were the results obtained in a valid and reliable manner? 8. Was appropriate statistical analysis used? For overview studies, we referred to the AMSTAR II (Appraisal of Guidelines for Research and Evaluation II) to evaluate the scope and purpose, participants, rigorous, clarity, applicability, and independence of the included overview studies [15]. The following evaluation criteria were used: the overall assessment results of the comprehensive evaluation included inclusion, exclusion, or requirement for more information. The quality of the literature was separately evaluated by two researchers. In the event of disagreement, a third researcher was consulted to obtain consensus.

### 2.7. Systematic Review

A systematic review was performed to assess the problems in dissemination of acupuncture-moxibustion clinical practice guidelines among clinical practitioners.

## 3. Results

### 3.1. Literature Screening Process and Results

**Figure 1:**
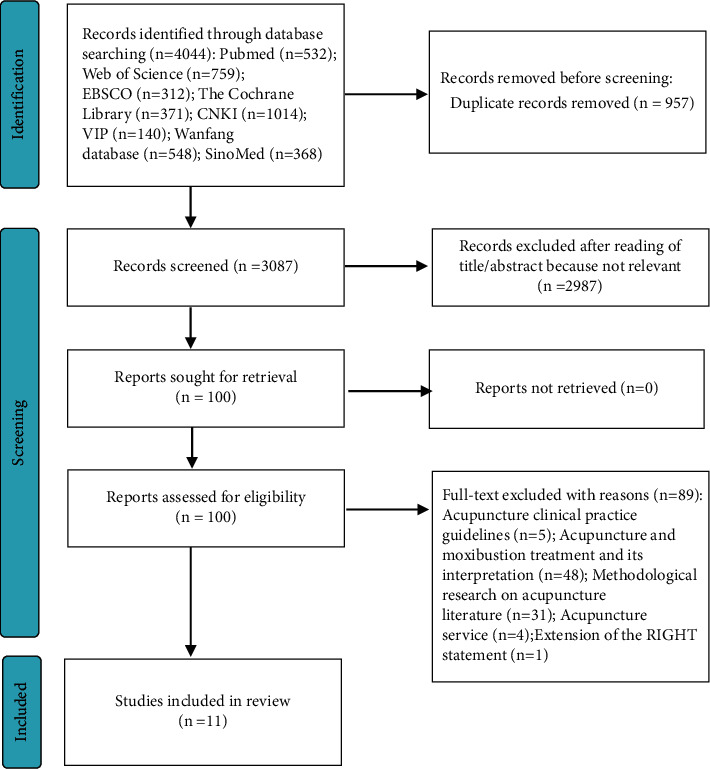
Literature screening process and results.

An initial review of 4,044 relevant sources was conducted. After removing duplicates, 3,087 studies were identified. After screening the titles and abstracts, 100 studies were retained. After browsing the full-text articles, we further excluded 89 records. A final total of 11 studies [10, 13, 16–24] were reviewed (Figure 1).

### 3.2. Basic Characteristics and Quality Evaluation of the Included Studies

The basic characteristics of the included studies are shown in Table 1. Of studies, 11 (100%) [10, 13, 16–24] were from China, and the earliest [20] was published in 2014. Seven (63.6%) [10, 13, 17–20, 23] studies were from the Chinese Academy of Traditional Chinese Medicine and four were from Nanjing University of Traditional Chinese Medicine [21], Tianjin University of Traditional Chinese Medicine [22], Beijing University of Traditional Chinese Medicine [16], and Hubei University of Traditional Chinese Medicine [24]. Six studies (54.5%) [10, 16–18, 20, 23] were from the journal Chinese Acupuncture and Moxibustion, one (9.1%) [22] was a symposium proceeding, one (9.1%) [24] was published in the Chinese Journal of Integrative Medicine, two (18.2%) [19, 22] were master's theses and one [13] was a doctoral dissertation. Nine studies (81.8%) [13, 16, 18–24] were cross-sectional surveys and two (18.2%) [10, 17] were overviews of systematic reviews. The evaluated guidelines were different in each study: seven (63.6%) [13, 16, 19–23] were practice guidelines for a specific disease, one study (9.1%) [19] evaluated clinical practice guidelines for acupuncture therapies such as moxibustion and fire acupuncture, one (9.1%) [24] examined acupuncture guidelines and recommendations published in the United States, and two (18.2%) [13, 17] did not provide a description of the content of the evaluated guidelines. The results of the quality evaluation of the included studies are shown in Table 2 and Table 3. All studies were included in the systematic review.

AMSTAR II: Appraisal of Guidelines for Research and Evaluation II.

### 3.3. Problems in the Dissemination of Acupuncture Guidelines to Clinical Practitioners

Because of the different evaluation indicators used in the included studies, we evaluated 10 aspects of the clinical guidelines and problems in dissemination to practitioners identified in the included studies: clinical events defined by the guidelines, target population, application setting, health economics evaluation, content of recommended protocols, association of recommended protocols with evidence, association of recommended protocols with clinical practice, acupuncture-related practice norms, promotion methods and efforts, and methods and theories of guideline writing.

Clinical events defined by the guidelines. A total of three studies [10, 17, 19] reported problems defining clinical events in clinical guidelines. All three studies mentioned unclear and inconsistent criteria for the clinical problems/diseases targeted by the guidelines [10, 17, 19], and one mentioned that the process of identifying clinical events in guidelines was unclear [10] and that the guideline diagnostic criteria were not applicable to the clinical setting [19].

Target population of the guideline. Five studies [10, 13, 17, 19, 20] reported problems defining the target population of the guidelines, two studies reported that the target population of the guidelines was unclear and that some guidelines did not differentiate between population subgroups [10, 13], and one study mentioned that the process of identifying the target population of the guidelines was unclear [10]. Regarding clinical practitioners and patients in the target population, two studies mentioned low trust in acupuncture guidelines among clinical practitioners [19, 20]; two mentioned that the guidelines did not take patients' wishes, preferences, and values into account [13, 20]; one study reported a lack of attention to patients' healthy lifestyles and self-care [17]; and one mentioned that the guidelines did not take into consideration doctor-patient interactions or patients' personality traits or allow for individualization [13, 20].

Application environment of the guidelines—three studies [16, 18, 19] indicated that the dissemination of guidelines may be affected by the regions in which they were applied. Two of these studies also mentioned that the focus on guidelines varies by region within a country [16, 18]; one mentioned that it was limited by local legislation and health insurance payment [18], and one mentioned that it may be affected by the way clinical practitioners learn to apply new knowledge, their habits, traditions, and preferences [19].

Health economics evaluation in the guidelines—four publications referred to the lack of guideline health economics evaluation supported by reliable data, such as cost-effectiveness ratios of recommended interventions [13, 21, 22, 24].

Content of the recommended programs in the guidelines—nine [10, 13, 16, 17, 19–22, 24] publications identified problems with the content of the recommended protocols in the guidelines; seven of these studies concluded that the guidelines generally did not state outcome indicators or indicators for evaluating the efficacy of interventions to determine the effectiveness of the treatment [10, 13, 17, 20–22, 24], and three studies concluded that the guidelines did not provide an analysis of the pros and cons of interventions [10, 13, 17]. Two studies reported that the acupuncture guidelines stated the strength of recommendations, but the process of reaching conclusions was unclear [13, 19], and four studies concluded that it was not possible to determine the difference in prognostic outcomes between strong and weak recommendations [10, 13, 19, 20]. Three studies reported that the description of the “content of recommendations” was unclear and that the “form of expression of recommendations” was unclear and could be improved [13, 16, 19]. One study concluded that the guideline recommendations did not clearly describe whether acupuncture could be used for both primary and comorbid conditions [13], and three studies concluded that the guidelines rarely considered the safety of acupuncture, including complications and adverse events [10, 22, 24].

Linking recommendations to evidence—four studies identified problems with the association between recommendations and evidence [10, 13, 16, 19]; two studies suggested that the guideline “process for developing recommendations” was unclear and that neither the process nor the rationale for developing recommendations was clear [10, 13]. Two studies suggested that the “association between recommendations and supporting evidence” was not rigorous and that it was unclear how the evidence affected recommendations [9, 11]. One study considered that the guidelines did not describe updates sufficiently, did not present differences between current status and research evidence, and did not explain whether the temporal characteristics of recommendations allowed for revision, while noting that the types of research evidence included were confusing [13]. Two studies considered that the quality of evidence supporting recommendations was poor [13]. Two studies mentioned the poor quality of the evidence for the recommendations [16, 19].

Discrepancies between recommendations and clinical reality—four studies [13, 17, 19, 20] concluded that there were substantial discrepancies between guideline recommendations and clinical practice. For example, three studies considered the evaluated guidelines difficult to apply in practice and unlikely to address practical problems of real clinical concern [17, 19, 20]. One study considered that there was a lack of emphasis on the importance of the timing of acupuncture interventions [17], and one study considered that the recommendations were not in line with routine clinical practice, difficult to practice, or posed an underlying risk [19]. One study considered that the guidelines were not naturally integrated with modern medical examination and treatments [20]. One study considered that the guidelines did not describe the facilitators and hindrances associated with their practical application [13].

Code of practice related to acupuncture—three [17, 20, 24] studies mentioned the lack of guidance on acupuncture techniques and standardized practices in the guidelines, and one study also pointed out that specific treatment recommendations for selecting and matching acupuncture points and identifying diseases and symptoms should be reflected in the recommendations [20].

Modalities and intensity of promotion of the guidelines—five studies [13, 19, 20, 22, 23] reported low accessibility, inadequate promotion, and limited access to clinical practice guidelines for acupuncture, pointing out that guidelines often did not mention guideline access and related appendices or documents [13].

Methodology and theory for the preparation of the guideline. One study concluded that current guideline development methods were not applicable to acupuncture and that guideline developers lacked an appropriate understanding of the methods and content of acupuncture guideline development [19], such as a lack of in-depth or even erroneous understanding of the GRADE framework [13], problems with the content and presentation of acupuncture guidelines, and lack of clarity of guideline levels [19]. One study concluded that acupuncture clinical practice guidelines do not report members' specialties and that the titles and positions of the individuals who developed the guidelines were not clearly reported, which potentially limits the representation of experts in reviews of acupuncture guidelines. It was also reported that the lack of oversight or audit criteria for some guidelines, lack of information related to sponsorship and funds, lack of documentation and publication of conflicts of interest of guideline development team members, lack of description of publication dates, and failure to provide the limitations of current guidelines and recommendations for future research may all limit guideline dissemination to clinics [13].

## 4. Discussion

### 4.1. Summary of Findings

A total of 11 studies were included in this systematic review and evaluation of the quality of evidence for the dissemination of acupuncture clinical practice guidelines to clinical practitioners. The studies reported problems with the Clinical Practice Guidelines for Evidence-Based Acupuncture: Insomnia [17]; 35 Evidence-Based Acupuncture Clinical Practice Guidelines (Group Standards) published in China 2010–2021 [10]; Clinical Practice Guidelines for Acupuncture: Migraine [18]; 2019 publication of moxibustion therapy, fire acupuncture, cupping therapy, acupuncture and bloodletting, acupuncture and knife therapy, electroacupuncture, acupuncture point application Evidence-Based Clinical Practice Guidelines for Acupuncture [19]; Evidence-Based Clinical Practice Guidelines for Acupuncture: Diabetic Peripheral Neuropathy [13]; the 2011 publication of Evidence-Based Clinical Practice Guidelines for Acupuncture in Chinese Medicine [21]; 20 clinical practice guidelines (2019) for acupuncture (five on nervous system diseases; four on musculoskeletal system or connective tissue diseases; two on mental, behavioural, or neurodevelopmental disorders; two on digestive system diseases; two on endocrine, nutritional, or metabolic diseases; two on respiratory diseases; one on genitourinary system diseases; one on skin diseases; and one on ear or mastoid diseases) [22]; 35 acupuncture guidelines for low back pain [16]; and 39 acupuncture guidelines and 80 recommendations published in the USA [24]. We examined other issues regarding guideline dissemination to clinical practitioners; for example, one study described the innovative use of the concept of an evidence ecosystem cycle [11]. The research sources, research institutions, and publications of the included studies were relatively concentrated, and the overlap and credibility of the issues raised by each study were high.

### 4.2. Differences from Previous Studies

Owing to the inclusion of 5 studies published after 2019, when the 2019 acupuncture group standard “Acupuncture and Moxibustion Clinical Practice Guidelines Formulation and Evaluation Standards” (CAAM-2019 [001]) was issued by the Chinese Society of Acupuncture and Moxibustion [10], the present review supplemented and extended previous findings on the organization, personnel, and process of guideline formulation; the use of evidence quality evaluation; recommendation plan classification; methods of creating expert consensus; guideline formulation and content; and other dimensions of guideline development. A more comprehensive summary of the problems existing in the dissemination of clinical practice guidelines for acupuncture and moxibustion included a lack of standardization in acupuncture clinical practice guidelines in defining clinical events, unclear target population, mismatch between the guidelines and the application environment, lack of reliable health economics evaluation, poor quality content of recommendations, lack of linkage between recommendations and evidence, disassociation of recommendations from clinical practice, lack of acupuncture methodological guidance, limited transmission channels, and insufficient methodological and theoretical application in formulating guidelines. After many years of development, acupuncture is now widely used internationally; however, the technical level and clinical effects obtained by different clinical practitioners vary [25]. This current study showed a more systematic summary of how to improve the credibility, usability of acupuncture clinical practice guidelines, and standard methods for disseminating the guidelines to clinical practitioners. It will help the domestic and international acupuncture community to apply evidence that would facilitate appropriate decision-making about clinical events [3].

### 4.3. Recommendations for Acupuncture Clinical Practice Guidelines

It is recommended that acupuncture clinical practice guidelines more precisely define clinical events suitable for the target population [26, 27]. Clinical events, diagnostic criteria, treatment methods, and criteria for evaluating clinical effects should be defined. Patient preferences should be integrated, and international guideline standards should be developed. Guidelines should be suitable for practitioners in different regions and at different levels and address differences in the scope of application and key clinical events [28].

It is recommended that acupuncture clinical practice guidelines are relevant to clinical practice [26]. It is important to fully consider the characteristics of acupuncture clinical treatment and the complexity of acupuncture practice, and to not only rely on evidence but to also take into consideration the opinions of acupuncture clinicians and expert consensus. In-depth studies of common clinical acupuncture methods and therapies should be conducted. Guidelines should fit all roles of acupuncture in clinical practice. Guideline recommendation could be classified according to acupuncture methods and should include specific practice guidelines (e.g., for acupoint selection, stimulation methods, and acupuncture duration) [29]. Before a guideline is published, the opinions of clinical practitioners should be solicited to determine the clinical consensus on technical acupuncture skills, such as diagnosis, acupoint selection, use of acupuncture/moxibustion therapy, and other manipulative contents. Alternatively, clinical consistency tests could be conducted to test the development of acupuncture clinical practice guidelines. Clinical practice guidelines should be based on full consideration of the characteristics of the discipline of acupuncture and moxibustion, comprehensive evaluation of modern literature evidence, and integration of ancient literature and the experience of medical practitioners. Recommendations should be developed using appropriate clinical guidance. Attention should be paid to integration of guidelines with the results of modern tests and imaging examinations and to strengthening the study of health economics evaluation in the field of acupuncture and moxibustion.

There is a need to improve the evidence evaluation index system of acupuncture clinical practice guidelines. The selection of efficacy evaluation indicators and targeted data analysis should be combined with clinical needs to ensure the measurability of the recommendations. It is recommended that a detailed guideline update plan be developed and that targeted requirements are introduced into the guideline development process to promote greater clarity in the development process, greater methodological rigor in research, stronger associations, and more scientific evidence in the included studies. The clinical characteristics of the evidence should be based on real-world clinical problems that are of substantial interest or that need to be resolved. High-quality clinical trials appropriate to the practice of acupuncture and moxibustion, as well as multidisciplinary and interdisciplinary collaborative studies, would facilitate the development of guidelines that meet real clinical needs. It is also important to explore the methods of nonclinical trial findings, such as those found in ancient literature and the experiences of famous doctors, in evidence synthesis and other guideline aspects.

Guideline methodology recommendations are to further standardize the method and process of guideline formulation [30]. Close multidisciplinary collaboration, especially between methodologists and clinical experts, is needed. Methodologists should be involved throughout the guideline development process. The guideline development methodology could be enhanced by improving methodological quality control in clinical study design; reporting of members' specialties; clear reporting of titles, positions, and contributions of individuals to guideline development; oversight or audit criteria; information related to sponsorship and use of funds; publications and updates of practice; limitations of the current guidelines; pros and cons of interventions; and recommendations for future research [31]. To strengthen methodological research on guideline development for acupuncture practice, evidence-based acupuncture guidelines need to be reasonably informed by international guideline development methods, combined with a consideration of the unique characteristics of acupuncture clinical practice. The quality and completeness of guideline reporting could be improved by comparing the entries in guideline evaluation tools such as AGREE II and RIGHT to evaluate guidelines [32]. It would be useful to establish an acupuncture clinical practice guideline application-feedback platform to actively track, investigate, and collect clinical users' opinions and suggestions about guidelines. This would facilitate timely identification of problems in acupuncture guidelines and prompt appropriate updates.

## 5. Limitations

The limitations of this study were that all included studies were from China (no relevant literature was published outside China) and that the research institutions and researchers were limited to the Chinese Academy of Traditional Chinese Medicine and domestic traditional Chinese medicine universities. This may have led to publication bias and results bias. Additionally, all the included studies were reviews or cross-sectional surveys; there was a lack of studies using high-quality experimental designs.

## 6. Conclusions

There is an urgent need to improve the credibility, usability, and standardization of acupuncture clinical practice guidelines, to standardize and disseminate tools for analysing information to clinical practitioners, and to develop and publish credible guidelines to help the domestic and international acupuncture community to apply evidence. Recommendations for promoting the dissemination of acupuncture clinical practice guidelines are to define clinical events suitable for the target population, to be relevant to clinical reality, to improve the evidence evaluation index system, and to further standardize the method and process of acupuncture clinical practice guideline development. Future systematic reviews are needed that include new studies.

## Figures and Tables

**Table 1:** Basic characteristics of the included studies.

Study	Country	Research organization	Publication journal	Study design	Guidance evaluated	Evaluation methods/evaluation dimensions	Evaluator	Issues^*∗*^
Hu, Jing [17]	China	Institute of Acupuncture and Moxibustion, China Academy of Chinese Medical Science**s**	Chinese Acupuncture and Moxibustion	General narrative	Clinical Practice Guidelines for Evidence-Based Acupuncture: Insomnia	Guideline development: “Define the disease,” “Formulate the clinical problem,” “Formulate the recommended protocol”	Authors	1, 2, 3, 4, 5, 6, 7

Zhao, Nanqi [10]	China	Institute of Acupuncture and Moxibustion, China Academy of Chinese Medical Sciences	Chinese Acupuncture and Moxibustion	General narrative	35 Evidence-based Acupuncture Clinical Practice Guidelines (Group Standards) published in China 2010–2021	“Purpose and scope,” “Rigor of formulation,” “Clarity of expression,” “Applicability” of the evaluation guide	35 lead drafters of the guide	1, 3, 5, 8, 11, 10, 12. 13, 52

Hu, Jing [18]	China	Institute of Acupuncture and Moxibustion, China Academy of Chinese Medical Sciences	Chinese Acupuncture and Moxibustion	Cross-sectional survey	Clinical Practice Guidelines for Acupuncture: Migraine	Questionnaire: current status of acupuncture application abroad and demand for international standard development, priority of disease types for international standard development, need for international standard translation, key clinical issues to be addressed by international standards, correlation between professional level and demand intention	Top international experts in the field of acupuncture, clinical practitioners, experts in policy-making and acupuncture education, research institutions, experts in the field of acupuncture standards, experts in evidence-based medicine and methodology, patient representatives	14, 15

Guo, Lihua [19]	China	China Academy of Chinese Medical Sciences	Master's thesis	Cross-sectional survey	2019 publication of moxibustion therapy, fire acupuncture, cupping therapy, acupuncture and bloodletting, acupuncture and knife therapy, electroacupuncture, acupuncture point application Evidence-Based Clinical Practice Guidelines for Acupuncture	Questionnaire + n-depth interview: evaluation of guideline quality, barriers to application, and applicability of acupuncture clinical practice guidelines	Guideline developers, evaluators, and relevant researchers	1, 2, 9, 12, 13, 16, 17, 18, 19, 20, 21, 22, 23, 24, 25, 26, 27, 28

Chen, Chao [20]	China	Institute of Acupuncture and Moxibustion, China Academy of Chinese Medical Sciences	Chinese Acupuncture and Moxibustion	Cross-sectional survey	Not specified	Questionnaire: clinical use of acupuncture guidelines, clinical practitioners' perceptions of guidelines, clinical recourse, most needed clinical guidance, how to improve guideline implementability	Clinicians, university teachers, medical students	2, 3, 4, 13, 26, 27, 29, 30, 31, 32, 33

Chen, Chao [13]	China	China Academy of Chinese Medical Sciences	Doctoral dissertation	Systematic evaluation of health economics, cross-sectional surveys	Evidence-Based Clinical Practice Guidelines for Acupuncture: Diabetic Peripheral Neuropathy	Systematic evaluation: health economics evidence Tools for acupuncture: the AGREE II, RIGHT statement, GLIA evaluation of methodological quality, quality of reporting and implementability evaluation questionnaire: use of guidelines and their implementability issues	Author, clinical practitioner of acupuncture	8, 9, 10, 13, 34, 35, 31, 36, 37, 38, 39, 16, 40, 34, 41, 42, 43, 44, 45, 46, 47, 48, 49, 50, 17, 51, 3,

Chen, Hao [21]	China	Nanjing University of Chinese Medicine	Proceedings of the 2014 Annual Meeting of the Clinical Branch of the Chinese Society of Acupuncture and Moxibustion and the 21st National Clinical Symposium on Acupuncture and Moxibustion	Cross-sectional survey	the 2011 publication of Evidence-Based Clinical Practice Guidelines for Acupuncture in Chinese Medicine	AGREE II	Authors	50, 34, 3

Lingling, Zhang [22]	China	Tianjin University of Traditional Chinese Medicine	Master's thesis	Cross-sectional survey	20 clinical practice guidelines (2019) for acupuncture	RIGHT Statement, AGREE-China Tools	Two researchers	3, 52, 34, 26

Guo, Lihua [23]	China	Institute of Acupuncture and Moxibustion, China Academy of Chinese Medical Sciences	Chinese Acupuncture and Moxibustion	Cross-sectional survey	20 evidence-based clinical practice guidelines for acupuncture	AGREE II	Two researchers	26

Yang Xingyue [16]	China	School of Acupuncture-Moxibustion and Tuina, Beijing University of Chinese Medicine	Chinese Acupuncture and Moxibustion	Cross-sectional survey	35 acupuncture guidelines for low back pain	—	Authors	15, 22

Guo, Y [24]	China	College of Acupuncture and Orthopaedics, Hubei University of Chinese Medicine	Chinese Journal of Integrative Medicine	Cross-sectional survey	39 acupuncture guidelines and 80 recommendations published in the USA	AGREE II	Two researchers	4, 3, 52, 34

^*∗*^ Appendix 2. AGREE II: Appraisal of Guidelines for Research and Evaluation II; RIGHT, Reporting Items for practice Guidelines in HealThcare; GLIA, GuideLine Implementability Appraisal.

**Table 2:** Quality assessment of the included studies (cross-sectional study).

Study	Evaluation tools	1. Were the criteria for inclusion of evaluators clearly defined?	2. Were the guidelines and settings studied described in detail?	3. Was the evaluation of the guidelines performed in a valid and reliable manner?	4. Was the situation measured using objective criteria?	5. Were confounding factors affecting the practice of the guidelines identified?	6. Were strategies in place to address confounding factors?	7. Were the results obtained in a valid and reliable manner?	8. Was appropriate statistical analysis used?	Overall assessment
Hu, jing [18]	Evaluation transect study (JBI) inventory (last revised in 2017)	Yes	Yes	Not applicable	Yes	Unclear	Unclear	Yes	Yes	Inclusion
Guo, lihua [19]		Yes	Yes	Not applicable	Yes	Unclear	Unclear	Yes	Yes	Inclusion
Chen, chao [20]		No	No	Yes	Yes	Unclear	Unclear	Yes	Yes	Inclusion
Chen, chao [13]		Yes	Yes	Yes	Yes	Yes	Unclear	Yes	Yes	Inclusion
Chen, hao [21]		Yes	Yes	Yes	Unclear	Unclear	Unclear	Yes	Yes	Inclusion
Lingling, zhang [22]		Yes	Yes	Yes	Yes	Unclear	Unclear	Yes	Yes	Inclusion
Guo, lihua [23]		Yes	Yes	Yes	Yes	Unclear	Unclear	Yes	Yes	Inclusion
Duan, yutin [16]		Yes	Yes	No	No	Unclear	Unclear	Yes	Yes	Inclusion
Guo, Y [24]		Yes	Yes	Yes	Yes	Unclear	Unclear	Yes	Yes	Inclusion

JBI: Joanna Briggs Institute.

**Table 3:** Quality assessment of the included studies (overview studies).

Study	Evaluation tools	Domain 1 scope and purpose (%)	Domain 2 participants (%)	Domain 3 rigorous (%)	Domain 4 clarity (%)	Domain 5 applicability (%)	Domain 6 independence (%)	Overall assessment	Is it recommended
Hu, Jing [17]	AMSTAR II	72.2	58.3	65.6	80.6	52.1	8.3	3	Yes (used after revision)
Zhao, Nanqi [10]	AMSTAR II	69.4	38.9	26.0	80.6	39.6	8.3	5	Yes (used after revision)

## Supplementary Materials

Supplementary Materials Appendix 1: search strategy. Table 1: CNKI (1014). Table 2: VIP (140). Table 3: Wanfang (548). Table 4: SinoMed (368). Table 5: PubMed (532). Table 6: Web of Science (759). Table 7: EBSCO (312). Table 8: Cochrane Database (371). Appendix 2: problem description (52 items).
