# Supplementary material for: Dissemination of Acupuncture-Moxibustion Clinical Practice Guidelines among Clinical Practitioners: A Systematic Review of Quality Assessment Studies
Source: Evid Based Complement Alternat Med. 2022 Apr 7;2022:8334397. doi: 10.1155/2022/8334397 (PMC9068293; doi:10.1155/2022/8334397)
Supplement: Supplementary Materials — Appendix 1: search strategy. Table 1: CNKI (1014). Table 2: VIP (140). Table 3: Wanfang (548). Table 4: SinoMed (368). Table 5: PubMed (532). Table 6: Web of Science (759). Table 7: EBSCO (312). Table 8: Cochrane Database (371). Appendix 2: problem description (52 items). [file 8334397.f1.docx]

## Supplementary Materials

# Appendix 1. Search strategy

Table 1 CNKI (1014)

| STEPS | Search Terms (in Chinese) | |
| --- | --- | --- |
| 1 | | (TKA= acupuncture or acupuncture or milli needle or electroacupuncture or warm acupuncture or moxibustion or ear acupuncture or transcutaneous electrical stimulation or acupoint application or acupoint embedding line or heat-sensitive moxibustion) and (TKA = clinical practice guidelines or evidence-based guidelines or guidelines) |

Table 2 VIP (140)

| STEPS | Search Terms (in Chinese) |
| --- | --- |
| 1 | (Title or keyword = acupuncture or acupuncture or milli needle or electroacupuncture or warm acupuncture or moxibustion or ear acupuncture or transcutaneous electrical stimulation or acupoint application or acupoint embedding line or heat-sensitive moxibustion) and (Title or keywords = clinical practice guidelines or evidence-based guidelines or guidelines) |

Table 3 Wanfang (548)

| STEPS | Search Terms (in Chinese) |
| --- | --- |
| 1 | (Title or keyword = acupuncture or acupuncture or milli needle) or electroacupuncture or temperature acupuncture or moxibustion or ear acupuncture or transcutaneous electrical stimulation or acupoint application or acupoint embedding or heat-sensitive moxibustion) AND (Title or keyword = clinical practice guideline or evidence-based guideline or guideline)) |

Table 4 SinoMed (368)

| STEPS | Search Terms (in Chinese) |
| --- | --- |
| 1 | “acupuncture”[common field] or “acupuncture”[common field] or “fine needle”[common field] or “electroacupuncture”[common field] or “warm needle”[common field] or “moxibustion”[common field] or “auricular acupuncture”[common field] or “transcutaneous electrical stimulation”[common field] or “acupoint application”[common field] |
| 2 | “acupoint embedding”[common field] or “thermal moxibustion”[common field] or “fire needle”[common field] or “small needle knife”[common field] or “press needle”[common field] or “skin needle”[common field] or “intradermal needle”[common field] or “hand twist needle”[common field] or “triangular needle”[common field] |
| 3 | “scalp needle”[common field] or “eye needle”[common field] or “acupoint injection”[common field] |
| 4 | “clinical practice guidelines”[common field] or “evidence-based guidelines”[common field] or “guide”[common field] |
| 5 | (#3) or (#2) or (#1) |
| 6 | ((#5) and (#4)) and 2008-[date] |

Table 5 PubMed (532)

| STEPS | | Search Terms |
| --- | --- | --- |
| 1 | ((((((((((((((((((((((acupuncture[MeSH terms]) OR (acupuncture-moxibustion[Title])) OR (acupuncture-therapy[Title])) OR (filiform needle[Title])) OR (electroacupuncture[Title])) OR (warm needling[Title])) OR (acupuncture[Title] AND moxibustion[Title])) OR (moxibustion[Title])) OR (auricular needling[Title])) OR (transcutaneous electrical stimulation[Title])) OR (acupoint application[Title])) OR (acupoint catgut embedding[Title])) OR (thermal moxibustion[Title])) OR (fire-needle[Title])) OR (little needle-scalpel[Title])) OR (press-needle[Title])) OR (dermal needle[Title])) OR (intradermal needle[Title])) OR (manual acupuncture[Title])) OR (three-edged needle[Title])) OR (scalp acupuncture[Title])) OR (eye acupuncture[Title])) OR (acupoint injection[Title]) | |
| 2 | ((clinical practice guidelines[Title]) OR (evidence-based guidelines[Title])) OR (guidelines[Title]) | |
| 3 | ((((quality evaluation[Title/Abstract]) OR (quality control[Title/Abstract])) OR (implementation[Title/Abstract])) OR (applicability[Title/Abstract])) OR (evaluation[Title/Abstract]) | |
| 4 | 1 and 2 and 3 | |

Table 6 Web of Science (759)

| STEPS | | Search Terms |
| --- | --- | --- |
| 1 | ((((((((((((((((((((((TS=(acupuncture-moxibustion)) OR TS=(acupuncture-therapy)) OR TS=(acupuncture)) OR TS=(filiform needle)) OR TS=(electroacupuncture)) OR TS=(warm needling)) OR TS=(acupuncture and moxibustion)) OR TS=(moxibustion)) OR TS=(auricular needling)) OR TS=(transcutaneous electrical stimulation)) OR TS=(acupoint application)) OR TS=(acupoint catgut embedding)) OR TS=(thermal moxibustion)) OR TS=(fire-needle)) OR TS=(little needle-scalpel)) OR TS=(press-needle)) OR TS=(dermal needle)) OR TS=(intradermal needle)) OR TS=(manual acupuncture)) OR TS=(three-edged needle)) OR TS=(scalp acupuncture)) OR TS=(eye acupuncture)) OR TS=(acupoint injection) | |
| 2 | ((TS=(clinical practice guidelines)) OR TS=(evidence-based guidelines)) OR TS=(guidelines) | |
| 3 | ((((TS=(quality evaluation)) OR TS=(quality control)) OR TS=(implementation)) OR TS=(applicability)) OR TS=(evaluation) | |
| 4 | #1 AND #2 AND #3 | |

Table 7 EBSCO (312)

| STEPS | Search Terms |
| --- | --- |
| 1 | (+acupuncture-moxibustion+OR+acupuncture-therapy+OR+acupuncture+OR+filiform+needle+OR+electroacupuncture+OR+Warm+needling+OR+Acupuncture+and+Moxibustion+OR+Moxibustion+OR+auricular+needling+OR+transcutaneous+electrical+stimulation+OR+Acupoint+application+OR+Acupoint+catgut+embedding+OR+Thermal+moxibustion+OR+fire-needle+OR+little+needle-scalpel+OR+press-needle+OR+dermal+needle+OR+intradermal+needle+OR+manual+acupuncture+OR+three-edged+needle+OR+scalp+acupuncture+OR+eye+acupuncture+OR+acupoint+injection+)+AND+(+Clinical+Practice+Guidelines+OR+Evidence-based+Guidelines+OR+Guidelines+)+AND+(+Quality+evaluation+OR+Quality+Control+OR+Implementation+OR+Applicability+OR+evaluation+) |

Table 8 Cochrane Database (371)

| STEPS | Search Terms |
| --- | --- |
| 1 | acupuncture-moxibustion OR acupuncture-therapy OR acupuncture OR filiform needle OR electroacupuncture OR warm needling OR acupuncture and moxibustion OR moxibustion OR auricular needling OR transcutaneous electrical stimulation OR acupoint application OR acupoint catgut embedding OR thermal moxibustion OR fire-needle OR little needle-scalpel OR press-needle OR dermal needle OR intradermal needle OR manual acupuncture OR three-edged needle OR scalp acupuncture OR eye acupuncture OR acupoint injection |
| 2 | clinical practice guidelines OR evidence-based guidelines OR guidelines |
| 3 | quality evaluation OR quality control OR implementation OR applicability OR evaluation |
| 4 | #1 AND #2 AND #3 |

# Appendix 2. Problem description

1 The clinical problem/target was not clear, and the standards were not uniform

2 Poor practicability, difficult to solve practical problems of high clinical concern

3 Lack of evaluation of curative effect outcome indicators of interventions

4 Lack of acupuncture techniques and standardized operation guidance

5 It was difficult to balance innovation and universality

6 Lack of emphasis on the importance of the timing of acupuncture intervention

7 Lack of attention to the patient’s own healthy lifestyle and self-care

8 Unclear target population (such as subgroup population)

9 The “relation between recommendations and supporting evidence” was not rigorous enough, and it was not clear how the evidence affects the recommendations

10 The “procedure for making recommendations” was insufficiently clear, and the link between the evidence and the guidelines was not clear

11 The process of determining “clinical problems” and the “target population” was unclear

12 The “recommendation content” and the “form of expression of the recommendations” were unclear

13 Unable to judge the difference in prognostic outcome of strong and weak recommended programs

14 Restricted by legislation and medical insurance payment

15 Different countries and regions have different focus on the guidelines

16 Recommendations in the guideline were not clearly described

17 The explanation of strong and weak recommendations in the guide was not clear

18 The diagnostic criteria in the guidelines do not apply to clinical practice

19 The recommended solution was difficult to operate or dangerous (problem)

20 Recommendations do not conform to routine clinical practice

21 The level of guidance was not clear

22 Poor quality of evidence supporting recommendations

23 The current general guideline formulation method was not suitable for acupuncture

24 Lack of scientific understanding of the methods and contents of the guidelines for acupuncture and moxibustion

25 Inaccessible guideline format and introduction

26 Promotional intensity and limited channels

27 Clinical practitioners have low trust in acupuncture guidelines

28 Application environment of the acupuncture guideline (methods, habits, traditions, and preferences for acquiring new knowledge)

29 The guidelines were not naturally integrated with modern medical examinations and treatments

30 No analysis of the pros and cons of interventions

31 No consideration of the patient’s wishes and values

32 The interaction between doctors and patients was not considered

33 Lack of syndrome differentiation and guidance on selecting acupoints

34 Lack of health economics evaluation of reliable data on interventions

35 The Acupuncture Clinical Practice Guide did not report the member’s profession

36 The types of research evidence included in the guidelines were confusing

37 Insufficient understanding (or even errors) of GRADE

38 Insufficient representation of review experts for acupuncture guidelines

39 The guideline did not adequately describe updates, and did not mention the difference between current status and research evidence

40 The guideline did not describe the factors that facilitate and hinder application

41 The guideline lacked oversight or auditing standards

42 Lack of information on sponsorship and use of funds

43 The guideline lacked documentation and publication of conflicts of interest of the members of the guideline development team

44 The guideline did not describe the year of publication

45 The titles and positions of the individuals who developed the guideline were not clearly reported

46 No mention of methods by which to obtain guidelines, and related attachments or documents

47 Limitations of current guidelines and recommendations for future research were not provided

48 Recommendations were not clear on whether the guideline can be used for both the main symptom and comorbidities

49 All guideline recommendations are associated with only one condition, and the logical relationships between the conditions are unclear

50 There was no explanation of the benefits and disadvantages of using the recommendations

51 Failure to consider the patient’s personality characteristics or allow individualization

52 Little consideration was given to the safety of acupuncture and moxibustion, which was related to complications and adverse events.
